# Supplementary figures and images for: A Reproducible Deep-Learning-Based Computer-Aided Diagnosis Tool for Frontotemporal Dementia Using MONAI and Clinica Frameworks
Source: Life (Basel). 2022 Jun 23;12(7):947. doi: 10.3390/life12070947 (PMC9323676; doi:10.3390/life12070947)

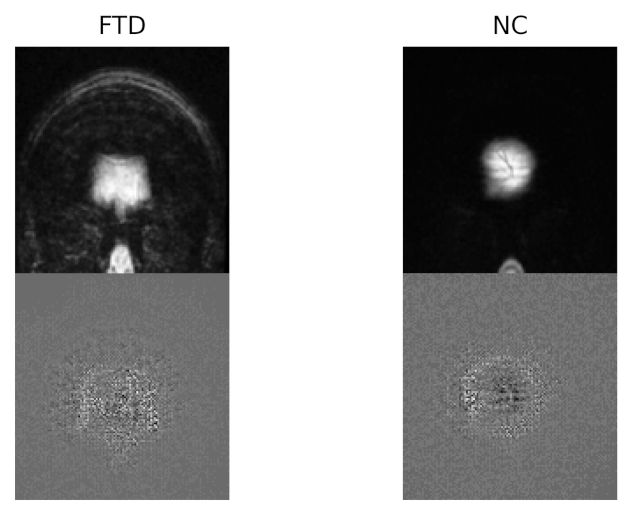

Supplement: Supplementary file 1 [file life-12-00947-s001.zip › animation s1.gif]
